# Exercise protects against methamphetamine-induced aberrant neurogenesis

**DOI:** 10.1038/srep34111

**Published:** 2016-09-28

**Authors:** Minseon Park, Harry Levine, Michal Toborek

**Affiliations:** 1 Department of Biochemistry and Molecular Biology, Miller School of Medicine at University of Miami, Miami, FL 33136, USA; 2 Jerzy Kukuczka Academy of Physical Education, Katowice, Poland

## Abstract

While no effective therapy is available for the treatment of methamphetamine (METH)-induced neurotoxicity, aerobic exercise is being proposed to improve depressive symptoms and substance abuse outcomes. The present study focuses on the effect of exercise on METH-induced aberrant neurogenesis in the hippocampal dentate gyrus in the context of the blood-brain barrier (BBB) pathology. Mice were administered with METH or saline by i.p. injections for 5 days with an escalating dose regimen. One set of mice was sacrificed 24 h post last injection of METH, and the remaining animals were either subjected to voluntary wheel running (exercised mice) or remained in sedentary housing (sedentary mice). METH administration decreased expression of tight junction (TJ) proteins and increased BBB permeability in the hippocampus. These changes were preserved post METH administration in sedentary mice and were associated with the development of significant aberrations of neural differentiation. Exercise protected against these effects by enhancing the protein expression of TJ proteins, stabilizing the BBB integrity, and enhancing the neural differentiation. In addition, exercise protected against METH-induced systemic increase in inflammatory cytokine levels. These results suggest that exercise can attenuate METH-induced neurotoxicity by protecting against the BBB disruption and related microenvironmental changes in the hippocampus.

Methamphetamine (METH) abuse is one of the fastest growing drug problems, with over 35 million users worldwide. METH popularity is due to its availability, easiness of use, low price, and high potential of addiction^1^. METH is known to alter the dopaminergic system (reviewed in^2^) and irreversibly damage neural and non-neural brain cells, which contribute to the development of neurological and psychiatric abnormalities in abusers. Recent studies indicated a strong impact of METH on the hippocampus, as METH abusing patients are characterized by decreased volume and maladaptive plasticity of the hippocampus^3^. In addition, hippocampal atrophy and the impairment of hippocampal-dependent memory tasks were described in such individuals^4^.

The hippocampal dentate gyrus (DG) is an important site of adult neurogenesis, including the processes of formation, survival, and integration of newly born neurons into the mature granule cell synaptic circuitry^5^. Evidence indicates that adult hippocampal neurogenesis is important for learning and memory and is affected by disease conditions associated with cognitive impairment, depression, or anxiety. With respect to drug addiction, correlative studies have demonstrated that METH abuse induces maladaptive plasticity in the hippocampus, such as loss of mature neurons and alterations of formation of neurons from neural stem cells (NSCs) and/or neural progenitor cells (NPCs). For example, it was demonstrated *in vitro* that METH exposure results in a dose-dependent reduction of proliferation of cultured rat hippocampal NPCs^6^. A repeated administration of METH to BALB/c mice showed decreased cell proliferation in the subventricular zone (SVZ) and the DG^7^.

The brain microvascular endothelium has also been shown to be a target of METH toxicity. METH exposure leads to intracellular ROS generation in cultured brain endothelial cells and disrupts the integrity of the blood-brain barrier (BBB), which is critical for brain homeostasis^8^. The BBB is enforced by tight junctions (TJs) between adjacent endothelial cells, which restrict the passages of substances from the blood to the brain^9^. Occludin, a 60–65 kDa transmembrane phosphoprotein, is highly expressed in cerebral endothelium, which binds to the two extracellular loops of claudin-5, a 20–25 kDa transmembrane protein, forming the paracellular component of the TJs. In addition, occludin is anchored to the actin cytoskeleton via binding to ZO-1, a peripheral protein^10^. Disruption of TJs has been associated with BBB disruption^11^,^12^, although a recent manuscript questioned this relationship^13^. The most significant alterations of the BBB integrity induced by acute exposure to METH (10 mg/kg) occur in the cortex and the hippocampus^8^,^14^. These changes are underlined by decreased TJ protein integrity in endothelial cells of brain microvessels. An accumulating body of evidence suggests that BBB disruption, followed by cerebral extravasation of circulating proteins including inflammatory molecules, may increase risk for the initiation and/or progress of cerebrovascular-based neurodegenerative disorders^15^,^16^.

NPCs are located in the hippocampus in close proximity with the microvessels^17^. Therefore, we hypothesized that METH-induced disruption of BBB may impair differentiation of NPCs to mature neurons, affecting neurogenesis. In addition, we employed voluntary exercise as the intervention to protect against METH-induced BBB permeability. Our results indicate for the first time that exercise can protect against chronic METH-induced impaired hippocampal neurogenesis by enhancing the BBB integrity and decreasing systemic levels of proinflammatory cytokines.

## Results

### Chronic METH administration alters TJ protein expression in brain microvessels

The main factor maintaining the homeostasis of the CNS is the proper function of the BBB, which is regulated by TJ proteins formed between adjacent endothelial cells. Therefore, we first determined the impact of chronic exposure to METH on TJ protein expression and localization. The analyses were performed 24 h after the last injection of METH.

**Figure 1:**
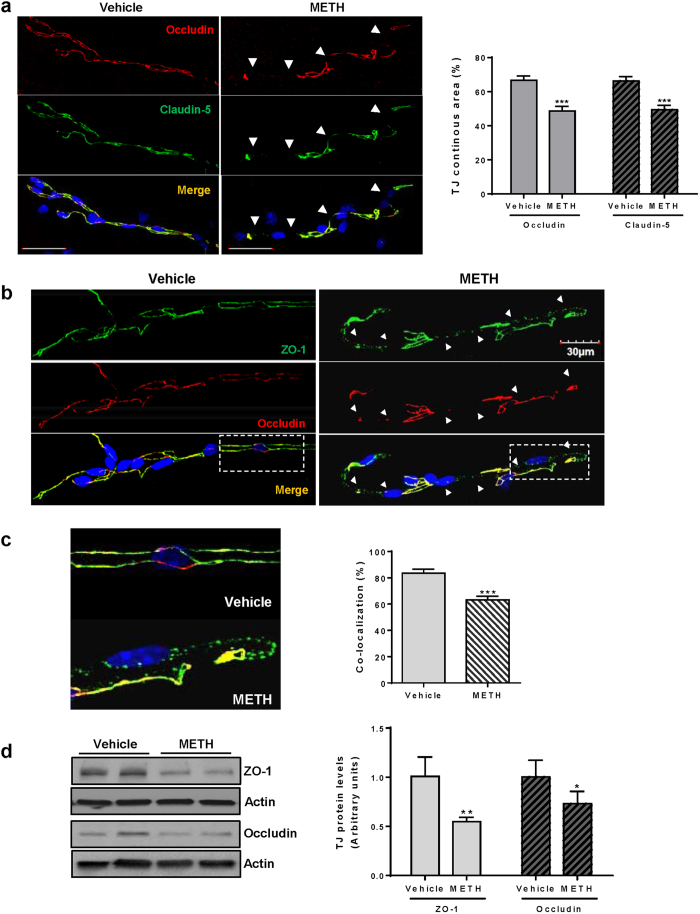
Chronic METH exposure alters TJ protein expression in brain microvessels. (**a**) Mice were exposed to METH or vehicle control as described in the Methods, followed by immunostaining of microvessels for claudin-5 (green) and occludin (red). Images were taken with a confocal microscope. Arrow heads indicate areas where occludin or claudin-5 immunoreactivity was discontinued. These areas were measured, expressed as percent of the total microvessel surface, and illustrated in the form of a bar graph. (**b**) Microvessels were isolated as in (A) and immunostained for ZO-1 (green) and occludin (red). Arrow heads indicate the areas where occludin and ZO-1 immunoreactivity was reduced and these proteins were not co-localized. (**c**) Enlarged images from B (rectangles) show the details of fragmented ZO-1 and occludin immunoreactivity in microvessels from controls and METH-exposed mice. The co-localization area of occludin and ZO-1 immunoreactivity was measured, expressed as percent of the total microvessel surface, and illustrated in the form of a bar graph. (**d**) Immunoblotting analysis of protein expression of occludin and ZO-1 in the hippocampal homogenates 24 h after chronic METH administration. *P < 0.05, **P < 0.01, or ***p < 0.0001 vs vehicle (n = 5 per group).

Control mice were characterized by a continuous and largely overlapping staining for claudin-5 and occludin throughout the length of microvessels (Fig. 1a, left panel). However, this uniform pattern was interrupted and fragmented (arrow heads) in microvessels from METH-exposed mice, resulting in over 25% reduction of staining continuity of both occludin and claudin-5 as compared to control group (Fig. 1a, right panel). Immunoreactivity of ZO-1 was also discontinued in microvessels from METH-exposed mice (Fig. 1b, arrow heads). These changes included a spot-like pattern of ZO-1 staining and are shown in more details in Fig. 1c (left panel). The merged regions of positive staining for both occludin and ZO-1 were quantified (Fig. 1c, right panel), confirming the loss of co-localization of these TJ proteins in METH-exposed mice. Such images are consistent with dislodged assembly and decreased integrity of TJs.

To further quantify the observed changes in TJ protein expression in the hippocampal regions, the hippocampi were dissected, homogenized, and the levels of ZO-1 and occludin were determined by immunoblotting. Actin was used as loading control. As shown in Fig. 1d, both ZO-1 and occludin expression was significantly decreased by METH exposure, corresponding to the results of immunoreactivity in the isolated microvessels (Figs 1b and 1c).

### METH administration disrupts the BBB integrity and increases hippocampal IL-1β levels

**Figure 2:**
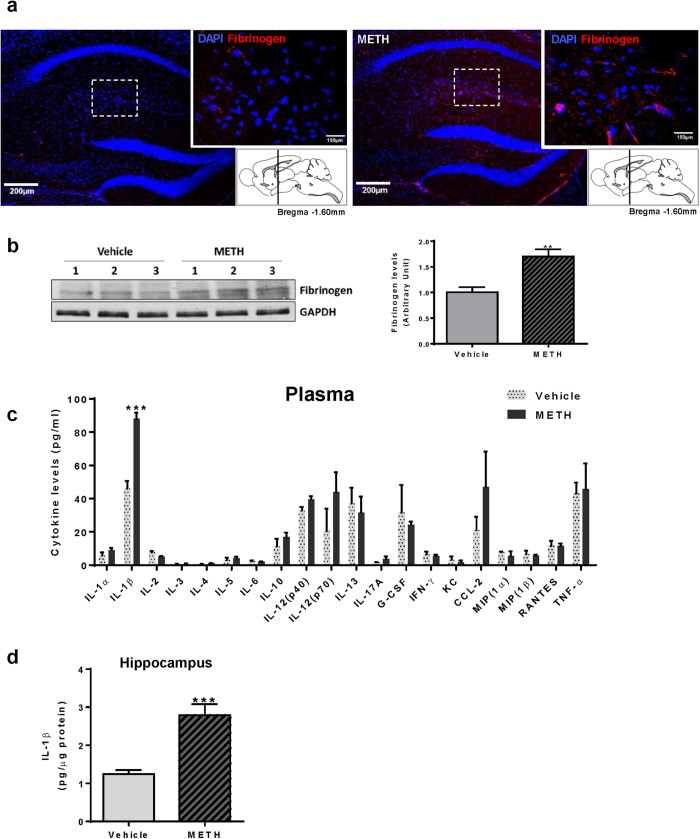
Chronic METH exposure increases BBB permeability and elevates hippocampal IL-1β levels. Mice were exposed to METH as in Fig. 1. All analyses were performed 24 h post METH administration. (**a**) Fibrinogen immunoreactivity in the cross sections of METH-exposed brains, indicating disruption of the BBB integrity in the hippocampus. Note that only minimal fibrinogen-positive staining was observed in control brains. Scale bar, 200 μm. (**b**) Fibrinogen levels in the hippocampal homogenates as determined and quantified by immunoblotting (n = 4 per group). (**c**) Plasma inflammatory cytokines as determined by Bio-Plex Pro Mouse Cytokine 23-plex assay kit. The levels of eotaxin, GM-CSF, and IL-9 were under detectable concentrations. Among determined cytokines, only IL-1β significantly increased after METH-administration (n = 6 per group). (**d**) Protein levels of IL-1β in the hippocampal homogenates (n = 8 per group). **p < 0.01 or ***p < 0.0001 vs vehicle.

Because expression and localization of TJ proteins were altered in the brain microvessels of METH-exposed mice, we next assessed whether these changes correspond to disrupted BBB integrity. BBB permeability in the hippocampal regions was evaluated by immunostaining for fibrinogen, a blood-born protein, which leaks into the CNS as the result of BBB disruption or vascular damage^18^. As indicated in Fig. 2a, immunoreactivity of fibrinogen was increased in the hippocampal sections from METH-exposed mice. These changes were confirmed and quantified by immunoblotting in the hippocampal homogenates, indicating statistical differences (Fig. 2b). These analyses were performed 24 h after chronic METH administration, which correspond to TJ disruption observed in Fig. 1.

Exposure to METH impacts a range of peripheral and central immune functions, including glial cell-mediated cytokine expression^19^. Therefore, we assessed the impact of METH administration on plasma levels of 23 different cytokines using multiplex bead-based ELISA. Among analyzed cytokines, only IL-1β levels were significantly increased in plasma samples from METH-exposed mice (Fig. 2c). While plasma IL-12 (p70) and CCL-2 levels were slightly elevated by chronic METH exposure, these changes were not statistically significant. Importantly, IL-1β protein levels were also significantly increased in the hippocampal regions in METH-exposed mice (Fig. 2d).

To discriminate whether IL-1β infiltrated the hippocampi via compromised BBB or was produced in the brain, we analyzed IL-1β mRNA expression by qPCR in the hippocampal regions. However, IL-1β mRNA was not detectible in this brain region both in control and METH-exposed mice (data not shown). These findings support the premise that chronic METH exposure increases plasma levels of IL-1β, which likely infiltrate the brain, including hippocampi, via disrupted BBB.

### METH administration induces abnormal neural differentiation in the hippocampus

NPCs are located in the hippocampal DG in a close proximity to brain microvessels, making this cell population vulnerable to BBB disruption^20^. Therefore, in the next series of experiments, neuronal differentiation was evaluated in this brain region in a model of chronic METH administration. Mice were sacrificed 24 h post the last injection with METH and the homogenates from the dissected hippocampi were analyzed for expression of NeuN (neuronal specific nuclear protein) and doublecortin (DCX, a microtubule-associated protein expressed in immature, i.e., migrating and differentiating neurons).

**Figure 3:**
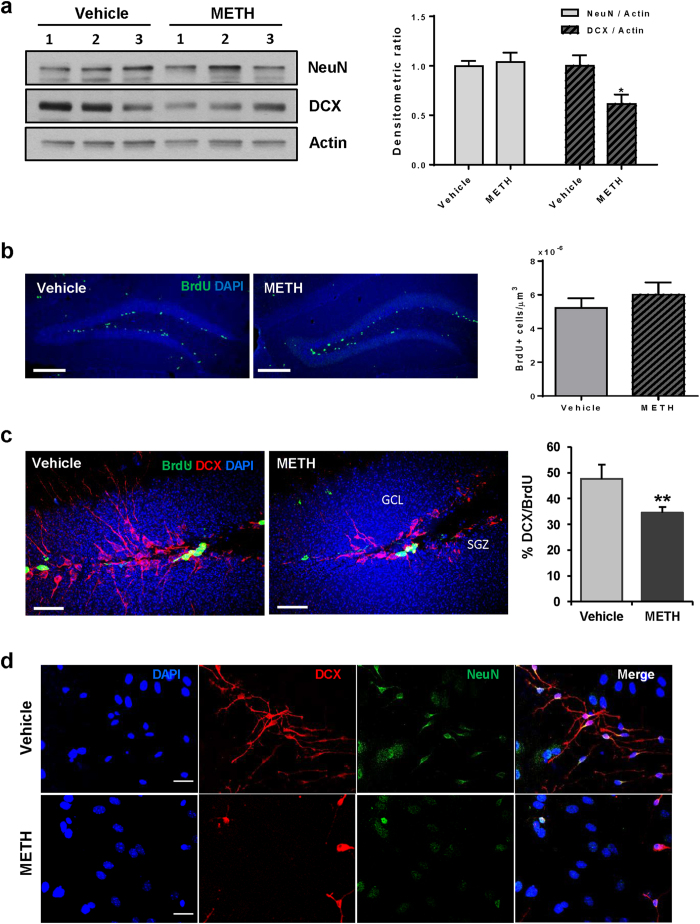
Neural differentiation is inhibited both *in vivo* and *in vitro* by chronic METH exposure. Mice were exposed to METH as in Fig. 1. All analyses were performed 24 h post METH administration. (**a**) The homogenates from dissected hippocampi were analyzed by immunoblotting for markers of immature (DCX, doublecortin) and mature neurons (NeuN, neuronal-specific nuclear protein). The images (left panel) show representative immunoblots and the quantitative results are presented as bar graph (right panel) (n = 3 per group). (**b**) Mice were i.p. injected with 150 μg/g of BrdU once a day for 5 days during METH or saline administration. Frozen sections (30 μm thick) were used for immunostaining with anti-BrdU antibody. The images (left panel) were captured by confocal microscope with a 10x objective. The BrdU-positive cells were counted in the hippocampal dentate gyrus (DG) and expressed as BrdU-positive cell numbers per volume (μm^3^) (right panel). Scale bar, 200 μm (n = 4–5 per group). (**c**) Using the sections prepared as in (B), newly formed, immature neurons were immunostained with anti-DCX and anti-BrdU antibodies (left panel). The percentage of double-positive cells compared to the total BrdU-positive cells was significantly reduced in METH-exposed mice (right panel). Scale bar, 30 μm (n = 4–5 per group). (**d**) Primary mouse neural stem cells were induced to differentiate in the presence of METH (10 μM) for 5 days followed by immunostaining for DCX (red) and NeuN (green). DAPI was used to stain nuclei (blue) and the images were captured with a fluorescence microscope. Scale bar, 20 μm *p < 0.05 or **p < 0.01 *vs* vehicle.

As shown in Fig. 3a, METH administration resulted in a significant reduction of DCX expression, whereas the levels of NeuN were not affected. Because DCX is expressed in type 1 and type 2 neuroblasts and in immature neurons during their differentiation^21^, we next evaluated whether METH treatment affects the formation of new neurons in the DG. The proliferating neural progenitor cells were labeled with BrdU (5-Bromo-2′-Deoxyuridine) throughout the METH exposure, followed by co-immunostaining of hippocampal slices for DCX and BrdU 24 h post the last dose of METH. The total number of BrdU-positive cells in the hippocampal DG was not changed between the control and the METH-exposed group (Fig. 3b).

As indicated in Fig. 3c, DCX immunoreactivity was strongly visible both in the subgranular zone (SGZ) and the granule cell layer (GCL) of the DG in control mice. In addition, DCX-positive cells expressed well developed apical processes that branched into the distal part of DG. A substantial population of DCX-positive cells was also immunoreactive for BrdU (Fig. 3c, left image). In contrast, the processes of the DCX-positive cells were visibly underdeveloped when compared to controls, and the ratio of DCX and BrdU-double positive cells to the total number of BrdU-positive cells was significantly decreased as the result of METH exposure (Fig. 3c, right graph).

The effects of METH on aberrant neural differentiation were confirmed in cultured primary mouse neural stem cells. A five day exposure to METH during their differentiation reduced the number of both NeuN and DCX immunoreactive cells and diminished the formation of DCX-positive processes (Fig. 3d). The combined *in vitro* and *in vivo* results suggest that chronic METH exposure inhibits neural differentiation in the hippocampus.

### Characterization of wheel running activity in post METH-administered mice

Physical exercise is known to promote cell survival and functional recovery after brain injuries^22^. We also reported that preceding voluntary exercise protected against METH-induced disruption of the BBB^14^. Therefore, we assessed the impact of exercise, in a form of voluntary wheel running, on METH-induced abnormal neural differentiation in the DG and altered TJ protein expression. This intervention was implemented after a 5 day METH exposure and lasted for 2 weeks. The control mice were placed in cages with a locked wheel.

**Figure 4:**
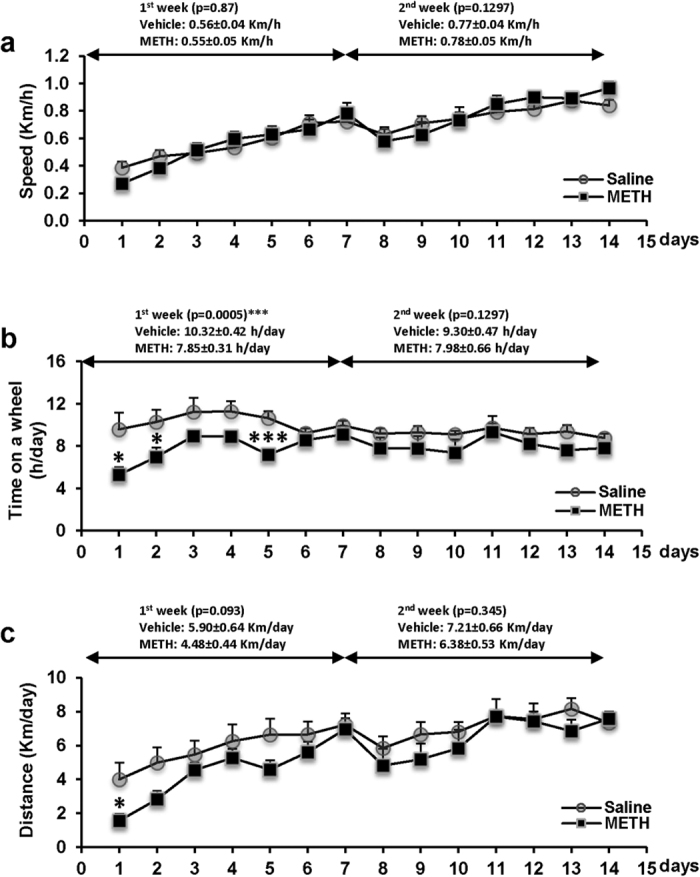
Characteristic of running activity in mice post chronic METH administration. Mice were exposed to METH as in Fig. 1. Then, a subset of mice was placed in cages with a free access to a running wheel 24 h after METH treatment discontinued (the exercise group). Control mice were placed in cages with locked wheels (the sedentary group). The running speed (**a**), time spent on the running wheel (**b**), and distance run (**c**) were recorded and daily records were plotted. *p < 0.05 or ***p < 0.001 vs saline control (n = 7 per group).

Both the post METH-exposed mice and the control mice in a similar fashion gradually increased their running speed over 2 weeks of voluntary exercise (Fig. 4a). However, post METH-exposed mice spent less time on running wheels in the first week of exercise period (7.85 ± 0.31 h/day) vs. control mice (10.32 ± 0.42 h/day). As the result, METH-exposed mice ran a significantly shorter distance than controls (4.48 ± 0.44 vs 5.90 ± 0.64 km/day, respectively) in the first week of exercise, (Fig. 4c). These differences disappeared over time, and average running time and distance were not significantly different between the groups in the second week of exercise. A small reduction in the running distance and speed between days 7 and 8 (Fig. 4a,c) was due to bedding change in the running cages.

### Exercise protects against post METH-induced alterations of TJ expression

**Figure 5:**
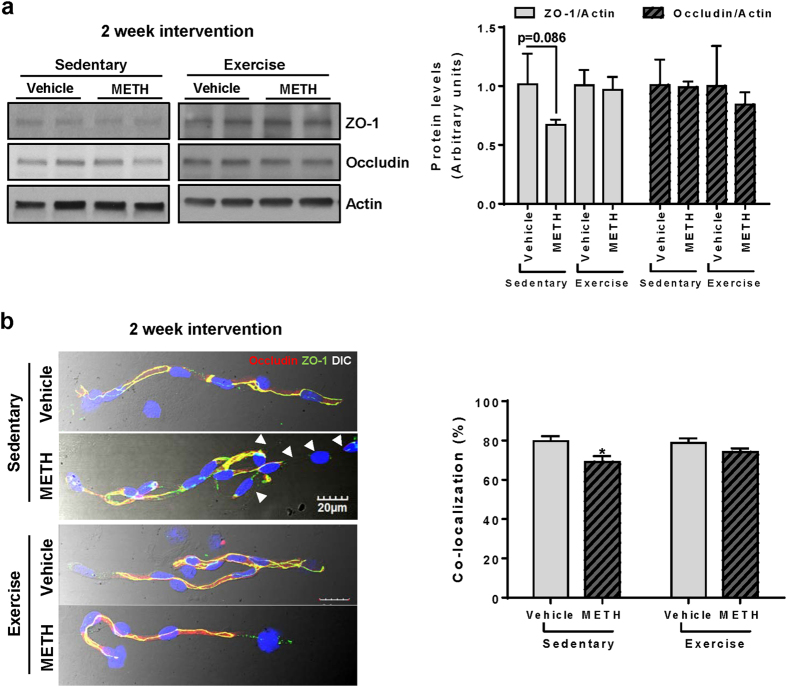
Exercise protects against chronic METH-induced alterations of TJ protein expression. Mice were exposed to METH as in Fig. 1, followed by a 2 week exercise or sedentary regimen. During exercise, mice did not receive any METH treatment. Analyses were performed at the end of exercise or sedentary period. (**a**) The homogenates from dissected hippocampi were analyzed by immunoblotting. Left panel, representative immunoblots; right panel, quantified densitometric data (n = 3 per group). (**b**) Isolated brain microvessels were immunostained for ZO-1 (green) and occludin (red) (left panel). Regions of co-localizations, depicted in yellow, were quantified, normalized to microvessel surface area, and expressed as a bar graph (right panel). *p < 0.05 *vs* vehicle.

To evaluate a long-term effects of METH on TJ proteins, we analyzed the total expression levels of ZO-1 and occludin in hippocampal homogenates two weeks after METH administration was discontinued, and mice were subjected to voluntary exercise regimen or remained sedentary. As indicated in Fig. 5a, the total levels of ZO-1 showed a tendency to be decreased in post METH-exposed sedentary mice, however, these changes were not statistically significant. In addition, occludin levels were the same in all experimental groups.

Because not only the levels of TJ proteins but also their assembly are critical for a proper BBB function, we next assessed ZO-1 and occludin immunofluorescence in microvessels isolated from post METH-exposed mice subjected to a 2 week of exercise or sedentary conditions. As shown in Fig. 5b (left, upper two images), microvessels from sedentary post METH-exposed mice expressed discontinuous immunoreactivity of these TJ proteins (arrow heads) even after 2 weeks post drug administration. In contrast, immunoreactivity and co-localization of ZO-1 and occludin expression levels returned to controls in post METH-exposed/exercised mice (left, lower two images). These results were confirmed by the quantitative analysis of the ratio of ZO-1 and occludin co-localization to the total microvessel surface (Fig. 5b; right graph). Overall, these results are consistent with the notion that exercise enhances BBB integrity by contribution to proper assembly of TJ proteins.

### METH-induced aberrant neurogenesis is attenuated by exercise

**Figure 6:**
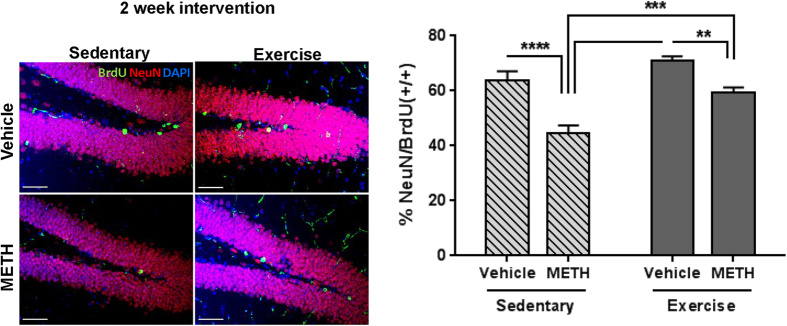
Exercise attenuates METH-induced aberrant neurogenesis. Mice were exposed to METH as in Fig. 1, followed by 2 weeks of exercise or sedentary conditions as in Fig. 4. Proliferating cells were labeled with BrdU during METH exposure as in Fig. 3b. Frozen brain slices were immunostained for NeuN (red) and BrdU (green) to detect newly differentiated neurons. Representative images are shown on the left panel and the percentage of double positive NeuN and BrdU cells (+/+) to the total number of BrdU positive cells are shown on the right panel. Scale bar  =  50 μm, **p < 0.01, ***p < 0.001, and ****p < 0.0001 (n = 4 per group).

Because a 2-week voluntary exercise regimen enhanced the recovery of TJ protein expression in microvessels from post METH-exposed mice, we next evaluated its impact on METH-induced aberrant neural progenitor differentiation in the DG. The hippocampal slices from post METH-exposed and vehicle-exposed controls mice (both exercised and sedentary) were co-immunostained for NeuN and BrdU. Because BrdU incorporation was performed during METH exposure, the ratio of NeuN/BrdU double positive cells to the total number of BrdU positive cells represent newly formed neurons which differentiated specifically during this time frame. This ratio was significantly reduced in sedentary post METH-exposed mice (Fig. 6). However, exercise significantly attenuated this effect, indicating partial recovery from METH-induced aberrant neurogenesis.

### Exercise protects against systemic induction of inflammatory cytokines by METH

Disruption of the barrier function promotes neuroinflammatory responses by allowing paracellular infiltration of inflammatory cells or molecules into the brain, contributing to impaired neural differentiation. In Fig. 2c,d, we showed that chronic METH exposure increases plasma and hippocampal levels of IL-1β. Therefore, we next assessed the impact of exercise intervention on plasma levels of the same inflammatory cytokines as those evaluated in Fig. 2.

**Figure 7:**
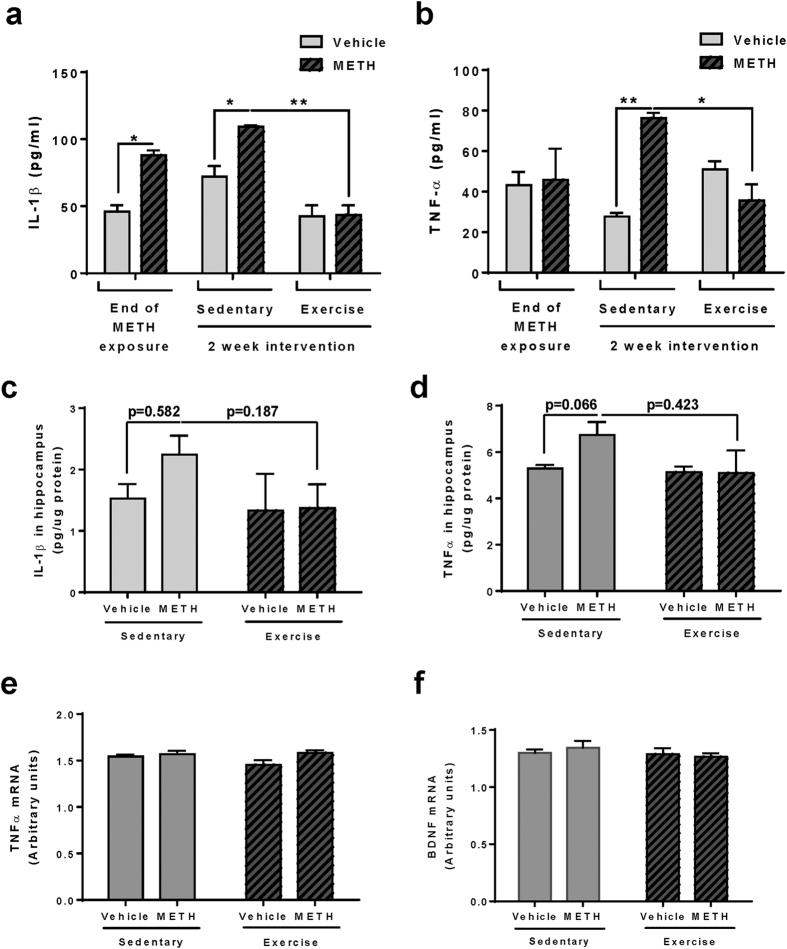
Exercise protects against METH-induced inflammatory cytokines in plasma. Mice were exposed to METH as in Fig. 1, followed by a 2 week exercise or sedentary regimen. Plasma IL-1β (**a**) and TNF-α (**b**) levels were measured by multiplex cytokine bead-based ELISA. First blood collection was performed 24 h post chronic METH or vehicle treatment and the second after the subsequent 2 weeks of exercise or sedentary housing. *p < 0.05 and **p < 0.01 (n = 6 per group). Protein levels of IL-1β (**c**) and TNF-α (**d**) in the hippocampal homogenates were measured as in (a and b). mRNA levels of TNFα (**e**) and BDNF (**f**) in the dissected hippocampi as measured by qPCR with mouse-specific primers and probes (n = 5 per group).

Among the studied cytokines, only levels of IL-1β and TNFα were affected by the employed treatment. While plasma IL-1β levels remained elevated in sedentary mice (Fig. 7a, Sedentary), a 2 week exercise regimen significantly attenuated this effect, bringing METH-induced IL-1β levels to the control concentrations (Fig. 7a, Exercise). Plasma TNF-α levels were in the range of control values at the end of METH administration (Fig. 7b); however, they subsequently increased significantly in post METH-exposed sedentary mice (Fig. 7b, Sedentary), suggesting ongoing inflammatory processes even after METH treatment was discontinued. Importantly, exercise effectively protected against upregulation of systemic TNF-α levels (Fig. 7b, Exercise).

Next, we analyzed the protein levels of these cytokines in the hippocampal homogenates. Both IL-β and TNFα were slightly, but not significantly, elevated in this brain region in post-METH/sedentary mice compared to the corresponding vehicle controls (Fig. 7c,d, Sedentary). A 2-week exercise attenuated these effects, bringing IL-1β and TNFα levels to the control concentrations (Fig. 7c,d, Exercise). Hippocampal TNFα mRNA expression was the same in all experimental groups and was not altered by exercise or sedentary conditions (Fig. 7e). IL-1β mRNA levels in the hippocampal regions were too low to be detected by 40 cycles of PCR reaction (data not shown).

METH is known to activate astrocytes^23^ and can induce microgliosis; therefore, we also evaluated the possible involvement of these events in the observed effects. However, both mRNA and protein expression of GFAP and Iba1 were not changed in any experimental groups. While BDNF has also been shown to play a neuroprotective role and impact cognitive functions^24^, its mRNA levels were not affected by the employed METH treatment and/or exercise (Fig. 7f). Overall, these results suggest that IL-1β and TNF-α can be the mediators that interfere with the neural differentiation in the DG. In addition, exercise may protect against METH-induced impaired neural differentiation by reducing the induction of inflammatory cytokines and increasing the expression level of TJ proteins in the capillary endothelium.

## Discussion

Chronic METH abusers generally initiate drug use by taking small amounts at variable intervals, followed by a gradual increase in doses. Although the diffusion time of METH into the circulation and its plasma levels depend on the route and amount of METH administration, the onset of peak effects in abusers occurs within 15 min post METH uptake and then gradually declines, with the half-life of 10–12 h in plasma^25^,^26^. In contrast, the half-life of plasma METH in rodents is about 60–70 min^27^; however, the residual levels of METH and its metabolite, amphetamine, remain elevated in the brain 4 h post exposure^28^. Due to a relatively short half-life and faster elimination rate, rodents do not accumulate METH after one day^29^. Therefore, our model was based on multiple daily administration of METH with escalating doses for 5 days. Mice are susceptible to the development of METH-induced neurocognitive dysfunctions as reported in the literature^30^,^31^.

Studies suggest that the BBB is one of the primary targets of METH toxicity. METH can alter BBB function through a direct impact on cerebrovascular endothelial cells and/or indirectly affecting the brain endothelium. For example, METH-induced oxidative stress is known to result in disruption of TJ integrity of brain microvascular endothelial cells, the effect that can be prevented by antioxidant treatment^32^. Using the escalating dose regimen, we demonstrated in the present study that METH exposure decreases the protein expression of occludin, claudin-5, and ZO-1 in the hippocampus and alters their co-localization in the brain microvessels, resulting in increased BBB permeability (Fig. 1).

Unlike other brain regions, microenvironmental changes induced by METH in the hippocampal DG can directly affect neurogenesis because of the localization of neural progenitor cells in the close proximity to hippocampal microvessels^33^. The brain microvasculature has been reported to be a key element of stem cell niches^34^. Indeed, selected parts of microvessels present in the SVZ lack astrocyte endfeet and amplifying progenitor cells are in direct contact with the brain endothelium at these sites^35^. It was suggested that the brain endothelium is an essential matrix and source of external cues for NPCs by creating microenvironment that mediates progenitor cell trafficking and differentiation^36^. Consistent with these observations, our study indicated underdeveloped apical processes of DCX-positive cells in the hippocampal DG and the reduced numbers of newly differentiating neurons as a result of chronic METH administration (Fig. 3c), suggest a link between METH-induced BBB disruption and the impairment of hippocampal adult neurogenesis.

While METH exposure reduced the number of differentiating neural cells in the DG (Fig. 3c), it did not affect the proliferation of neural progenitor cells (Fig. 3b). Thus, inhibition of hippocampal neurogenesis by METH can be attributed to impairment in differentiation of NPCs into the cells of neuronal lineage but not to alteration of NPCs proliferation. These results are in contrast to the report indicating that METH at 100 μM reduced proliferation of primary cultured rat NPCs^6^. However, that study employed very high concentrations of METH that exceed the typical plasma levels of METH abusers and was performed only *in vitro*. As METH abusers typically use 20–40 mg METH more than once a day^37^, METH body burden is estimated at ~50 mg, with the blood concentrations in the range of 0.1–11.1 μM^38^. The molar sum of METH plus amphetamine in the hippocampi of 14 METH abusers was between 2.3 and 32.3 nmol/g^39^. In rats self-administering METH for 28 days, the number of BrdU cells in the hippocampal DGs was significantly increased by METH exposure for 1 h twice weekly, not changed by daily self-administration for 1 h, and significantly decreased by daily exposure for 6 h^40^. These data indicate that METH has a complex impact on cell proliferation in the hippocampal DG, which differ according to METH concentration and mode of exposure.

We reported that exercise prior to acute METH administration can enhance antioxidative capacity at the level of brain capillaries and protect against disruption of the BBB integrity^14^. Therefore, we employed an exercise system based on wheel running in a model of chronic METH exposure in the present study. Importantly, exercise intervention was introduced post METH exposure in abstinent mice. Such an approach is critical from a translational point of view as exercise is frequently being implemented in dependency treatment of substance-abusing patients^41^,^42^. During the first week of METH abstinence, the exposed mice spent significantly less time on a running wheel as compared to the controls (Fig. 4b). However, these differences were reduced in the second week of exercise and both post-METH exposed mice and control mice run approximately the same distance with the same speed in the second part of our intervention study. Importantly, exercise exerted a significant protection against alterations of localization of occludin and ZO-1 in the microvessels (Fig. 5b). Such results denote enhanced integrity of TJs and the BBB functions and are consistent with literature reports. For example, treadmill running for 30 min per day for three days enhanced occludin expression and BBB functions as well as attenuated induction of MMP-9 in ischemic brain injury in rats^43^. In addition, exercised mice exhibited a reduction in brain metastasis formation, which was accompanied by a significant upregulation of occludin and claudin-5 protein expressions^44^.

Novel results reported in our study indicate that voluntary exercise significantly stimulated differentiation of neural progenitor cells to neuronal lineage in METH-exposed mice. It should be stressed out that exercise was implemented following METH exposure, indicating not only preventive but also a therapeutic impact. The importance of these findings stems from the fact that new hippocampal neurons are integrated into neuronal networks and play a key role in cognition^45^ and memory encoding by improving the resolution and correlation between new memories and old memories, which are encoded by mature neurons^46^. Our results are consistent with the notion that behavioral interventions, such as exercise^47^, dietary intervention^48^, or calorie restriction^49^, influence neural plasticity and enhance both neurogenesis and cognition. Indeed, physical exercise has emerged as a preventative strategy or treatment approach in neurodegenerative diseases related to aging^50^, binge ethanol exposure^51^, and METH dependence^41^. It was indicated that physical activity can enhance neurogenesis and improve Morris water maze performance of three-month-old female mice^47^. In humans, exercise was demonstrated to increase blood volume in the DG and improve cognitive score^52^.

**Figure 8:**
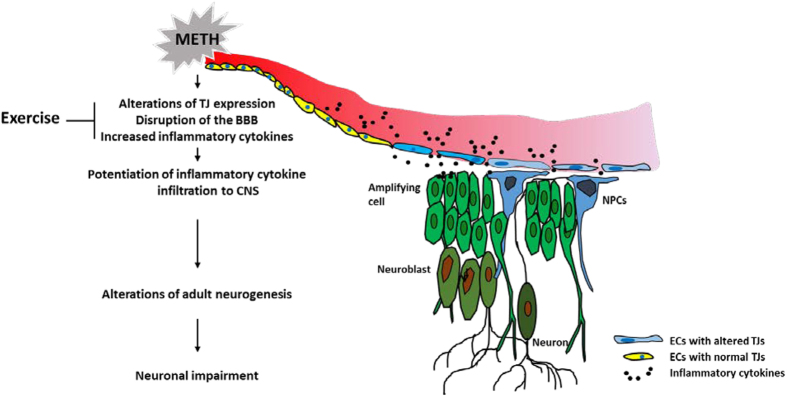
Proposed mechanisms of the protective effect of exercise on METH-induced alterations of hippocampal neurogenesis. METH disrupts TJ expression and localization in brain endothelial cells (ECs). In addition, METH increases the levels of inflammatory cytokines, such as IL-1β and TNF-α in plasma, which contribute to the disruption of the BBB integrity, leading to their entry into the brain. As a result of these events, differentiation of NPCs is impaired, which affects hippocampal neurogenesis. Exercise reverses these events by enhancing TJ protein expression and TJ assembly and by protecting against systemic induction of proinflammatory cytokines.

The underlying mechanisms of enhanced adult neurogenesis by physical activity are not fully understood. However, neurotrophins, such as BDNF, insulin-like growth factor I (IGF-I), and vascular endothelial growth factor (VEGF) have been recognized as potential mediators in this process^53^,^54^,^55^. Nevertheless, BDNF mRNA levels were not affected in the present study either by METH administration and/or exercise intervention (Fig. 7f). Similarly, the employed treatments did not affect astrogliosis and microgliosis as determined by lack of changes in mRNA and protein expression of GFAP and Iba1 in the hippocampus (data not shown). Overall, our results indicate the importance of anti-inflammatory pathways in exercise-mediated neuroprotection. Indeed, METH-induced levels of proinflammatory IL-1β and TNF-α were markedly attenuated in exercised mice (Fig. 7a–d). IL-1β has been shown to impair hippocampal neurogenesis via binding to its receptor present on proliferating and differentiated hippocampal NPCs^56^. TNF-α was demonstrated to be a negative regulator of progenitor cell proliferation^57^. Recent studies demonstrated that METH-treated macrophages *in vitro* increase levels of pro-inflammatory cytokine TNF-α^58^ and a 2-week self-administration of METH alters the frequency of CD4^+^ T cells and the pro-inflammatory cytokine production in a rat model^59^. Therefore, blood-borne IL-1β and TNF-α could be the mediators of METH-induced aberrant hippocampal neurogenesis after being transported across the disrupted BBB (Fig. 8).

In conclusion, this study demonstrates for the first time that METH-induced TJ alterations and the BBB disruption are concurrent to aberrant adult hippocampal neurogenesis in the DG. Importantly, physical exercise enhanced the expression of TJ proteins, stabilized the BBB, attenuated systemic inflammatory cytokine induction, and protected against METH-induced alterations in neurogenesis.

## Methods

### Animals, experimental groups, and isolation of brain microvessels

All animal procedures were approved by the University of Miami Institutional Animal Care and Use Committee in accordance with National Institutes of Health (NIH) guidelines and performed in accordance with the relevant guidelines and regulations. Male C57BL/6J mice (Jackson Laboratory, Bar Harbor, ME), 13 weeks of age were weight-matched and randomly assigned to various treatment groups. Mice were injected i.p. with METH (D-methamphetamine hydrochloride; U.S. Pharmacopeial Convention) three times per day for 4 days with an escalating dose regimen (starting with 0.2 mg/kg to the final dose of 2.4 mg/kg), using a step-wise increase of 0.2 mg/kg with each injection. On day 5, mice were administered i.p. with 4.0 mg/kg METH three times at 4 h intervals. Control mice were injected with saline as a vehicle. Similar models of escalating METH administration were used in the literature to reflect a pattern of drug intake by addicted users^60^,^61^. To label newly-proliferating cells, animals were i.p. injected with 150 μg/g of BrdU Roche, Indianapolis, IN) once per day for 5 days during the METH or vehicle exposure. At the time of METH and/or BrdU administration, mice were housed in standard conditions.

One set of mice was sacrificed 24 h post last injection with METH (or saline in the control mice), and the remaining mice were either subjected to voluntary wheel running (exercised mice) or remained in sedentary housing (the sedentary group) for the next 2 weeks. The length of exercise was chosen based on the fact that immature newborn granule cells enter an integrated stage as early as 2 weeks after being born. At this stage, the cells already express NeuN, a neuronal marker protein^62^,^63^.

Mice were housed individually in plastic cages containing a running wheel (Coulbourn Instruments, Whitehall, PA). Exercised mice had free access to the running wheel but sedentary mice were housed in cages with a locked wheel. Distance run, running speed, and time spent on a wheel were recorded individually for all mice using Clocklab software (Actimetrics, Wilmette, IL).

At the conclusion of METH treatment and/or exercise period, animals were anesthetized and perfused transcardially with saline. The brain microvessels were isolated from brains as described previously^8^. Briefly, brains were homogenized in cold isolation buffer (102 mM NaCl, 4.7 mM KCl, 2.5 mM CaCl_2_, 1.2 mM KH_2_PO_4_, 1.2 mM MgSO_4_, 15 mM HEPES, 25 mM NaHCO_3_, 10 mM glucose, 1 mM Na pyruvate) with proteinase inhibitors (Roche). Then, 26% dextran (MW, 150 kDa) in isolation buffer was added, mixed, and centrifuged (5,800 ×g; 4 °C) for 20 min. The supernatants were discarded; pellets were resuspended with the isolation buffer and filtered through a 100 μm nitrocellulose mesh filter. Filtered homogenates were re-pelleted by centrifugation (1,500 ×g; 4 °C) for 10 min and re-suspended in 200 μl of the isolation buffer.

### TJ protein assessment in brain microvessels

Freshly isolated microvessels were spread onto glass microscope slides and heat-fixed for 10 min at 95 °C, followed by 4% paraformaldehyde (PFA) for 15 min at room temperature. Samples were then washed three times with phosphate buffered saline (PBS) and permeabilized with 0.1% Triton X-100 in PBS for 10 min. Nonspecific binding was blocked with 10% normal goat serum (Abcam) in PBS for an hour and the microvessels were incubated with anti-ZO-1 antibody (Invitrogen) or anti-claudin-5 antibody (Invitrogen) overnight at 4  °C, followed by incubating with goat anti-rabbit antibody conjugated with Alexa Flour 488 (Invitrogen) for 2 hours at room temperature. Then, the microvessels were incubated with anti-occludin antibody conjugated with Alexa Flour 594 (Invitrogen) overnight at 4 °C. After three washings with PBS, slides were mounted with Vectashield HardSet Mounting Medium containing 4′,6-diamidino-2-phenylindole (DAPI) (Vector laboratory, CA). Images were acquired using a confocal microscope (Olympus Fluoview V5; Olympus America, Melville, NY). Microvessels sized between 4 and 8 μm in diameter were selected for analyses and at least 7 different microvessel images per each mouse sample were used for the quantification. The total area of analyzed microvessels from a mouse sample was between 12,000~26,000 μm^2^.

### Immunofluorescence

Mice were perfused transcardially with saline followed by 4% PFA, and the brains were collected. Following fixation with 4% PFA for 5 h and dehydration in 30% sucrose overnight, brains were sectioned (30 μm) on a cryostat. Subsequently, immunofluorescence was performed on free-floating brain sections. Sections were permeabilized in 0.2% Triton X-100 in PBS for 2 h, blocked in 10% normal goat serum in PBS for 2 h, and incubated with primary antibodies for 48 h. The following primary antibodies were used: rabbit anti DCX (1:200, Abcam), rabbit anti-NeuN (1:200, Abcam), mouse anti-fibrinogen β (1:200, Santa Cruz Biotechnology) or mouse anti-BrdU (1:200, Roche). The slides were then incubated with goat anti-rabbit-Alexa Fluor 568, goat anti-mouse-Alexa Fluor 568 secondary antibodies, or anti-mouse-Alexa Fluor 488 (1:200; Invitrogen) for 24 h, respectively. Afterwards, slices were washed and mounted onto glass microscope slides with Vectashield HardSet Mounting Medium containing DAPI (Vector laboratories). Images were acquired using a confocal microscope (Olympus) with z-series.

### Immunoblotting

Individual hippocampi were homogenized in RIPA buffer (50 mM Tris-HCl, 150 mM NaCl, 1% NP-40, 0.5% sodium deoxycholate, and 0.1% SDS, pH 7.4) supplemented with protease inhibitor cocktail tablets (Roche). Homogenates were centrifuged at 14,000 × *g* for 15 min and the supernatants were used for immunoblotting. Protein concentrations were determined using BCA Protein Assay Kit (Thermo Scientific, Rockford, IL). Samples were separated on 4–15% SDS-PAGE and transferred onto PVDF membranes (Bio-Rad Laboratories, Hercules, CA). Membranes were blocked for 1 h at room temperature in 3% (w/v) bovine serum albumin (BSA) in TBST buffer (50 mM Tris-Cl, pH 7.6; 150 mM NaCl; 0.1% (v/v) Tween-20), and incubated overnight at 4 °C with the respective antibodies as follows: rabbit anti-ZO-1 (1:1000, Invitrogen), mouse anti-occludin (1:1000, Invitrogen), rabbit anti-DCX (1:1000, Abcam), mouse anti-NeuN (1:1000, Millipore), mouse anti-fibrinogen β (1:1000, Santa Cruz Biotechnology) and monoclonal anti-β-actin-peroxidase (1:50000; Sigma-Aldrich). Individual immunoblots were visualized by an ECL Western blot detection kit (Amersham Biosciences, Piscataway, NJ), the proteins of interest were quantified by ImageJ software (National Institutes of Health, Bethesda, MD), normalized to actin expression, and used for statistical analysis.

### Cultures of primary neural stem and progenitor cells

Neurospheres derived from the cells isolated from embryonic day 14 (E14) mouse cortex were purchased from STEMCELL Technologies (Vancouver, Canada) and cultured according to the technical manual provided by the company. All cells were used in less than fifth passage. Neurospheres were dissociated with ACCUTASE (STEMCELL Technologies), seeded on glass coverslips (2 mm × 2 mm) pre-coated with poly-D-lysine (100 μg/ml; Sigma) and laminin (15 μg/ml; Sigma) for 2 h each, and incubated overnight at 37 °C in proliferating culture medium, which contained 20 ng/ml of recombinant human epidermal growth factor (STEMCELL Technologies). The next day, the cells were washed with PBS and induced differentiation by incubating cells with Complete NeuroCult NSC Differentiation Medium for 5 days with or without 10 μM METH. To immunostain for DCX and NeuN, the cells on coverslips were fixed in 4% PFA for 15 min and permeabilized in 0.2% Triton X-100 in PBS for 10 min. Nonspecific binding was blocked with 3% BSA in PBS for 1 h. Coverslips were then incubated overnight at 4 °C in a humidified atmosphere with rabbit anti-DCX and mouse anti-NeuN antibody diluted at 1:200 in PBS containing 3% BSA. After three washes with PBS, the coverslips were incubated with goat anti-rabbit-Alexa Fluor 568 or goat anti-mouse-Alexa Fluor 488 (1:200; Invitrogen) for 1 h at room temperature. Afterwards, coverslips were washed and mounted onto glass microscope slides with Vectashield HardSet Mounting Medium containing DAPI (Vector laboratories). Images were acquired using a fluorescence microscope (Eclipse Ti; Nikon Instruments, Melville, NY).

### Multiplex cytokine bead-based enzyme-linked immunosorbent assay (ELISA)

To detect plasma levels of inflammatory cytokines, a mouse cytokine 23-plex assay (Bio-Rad) was used according to the manufacturer’s instructions with a Bio-Plex MAGPIX multiplex reader. The assay was performed in 96-well plate and the levels of cytokines were quantified in pg/ml.

### Quantitative real-time polymerase chain reaction (qPCR)

Total RNA (500 ng) extracted from individual hippocampi was reverse-transcribed into cDNA using random hexamer primer mix and SuperScript RT-PCR kit (Takara Bio, Japan). TaqMan Universal Master Mix II (Applied Biosystems, USA) was used in 20 μl reactions containing 1 μl cDNA and FAM-probed target primers (TaqMan Gene Expression Assays). qPCR was performed using ABI 7500 instrument (Applied Biosystems, USA), followed by melt-curve analysis to further verify specificity and well-to-well consistency of specific product generation. Changes in gene expression were calculated using ΔΔCt (where Ct is cycle number at threshold) analytical method that includes normalization against the housekeeping gene, actin.

### Statistical analysis

The data were analyzed using GraphPad Prism software and experimental treatments were compared pairwise with control treatments using two way ANOVA followed by Turkey’s multiple comparisons test or Student’s t test with significance value at *p* < 0.05. Data are mean ± SEM; n = 3–6 mice per group. Multiple brain or brain microvessel images were analyzed from individual mice.

## Additional Information

**How to cite this article**: Park, M. *et al*. Exercise protects against methamphetamine-induced aberrant neurogenesis. *Sci. Rep.* **6**, 34111; doi: 10.1038/srep34111 (2016).
